# Differences and structural weaknesses of institutional mechanisms for health research ethics: Burkina Faso, Palestine, Peru, and Democratic Republic of the Congo

**DOI:** 10.1186/s12910-018-0284-3

**Published:** 2018-06-15

**Authors:** N’koué Emmanuel Sambiéni

**Affiliations:** 1 Université de Parakou, Parakou, Bénin; 2 Laboratoires d’Etudes et de Recherches sur les Dynamiques Sociales et le Développement local (LASDEL), Antenne de Parakou, Parakou, Bénin

**Keywords:** Committee, Ethics, Research institutions, University, Research protocol

## Abstract

**Background:**

Regardless of national contexts, the institutions responsible for research ethics, founded on international regulations, are all expected to be structured and to operate in a common way. Our experience with several countries on different continents, however, has raised questions in this regard. This article examines the differences and structural weaknesses of ethics committees in four countries (Burkina Faso, Palestine, Peru, and the Democratic Republic of the Congo) where we have conducted the same socio-anthropological study in the field of reproductive health.

**Methods:**

In addition to recording our observations during field surveys for this study, we performed a documentary review and interviewed expert members of ethics committees, research participants, and researchers who had experience with requesting ethics approvals for research protocols in the field of social sciences and health.

**Results:**

The results of this study showed that, despite having the same mandate, the committees functioned differently, while they all exhibited the same weaknesses. Thus, the universalization and standardization of institutional conditions for applying ethical standards in research still present problems that are, at the very least, relevant.

**Conclusion:**

This study on ethics committees in four countries demonstrated the profound influence of context on the ways in which different institutions function and enforce regulations. In effect, in all social fields, every innovation is infused by its context.

## Background

The development of research, the emergence of human rights and democracy issues in low- and middle-income countries, and the progressive encompassing of health in development are leading to more requests for research authorizations for purposes that have social, privacy, and integrity implications for participants in clinical trials and other studies. For this purpose, health research ethics committees rely on international regulations that govern their operations regardless of the country and institution in which they are located [1]. Ethics in research is enshrined in international treaties (Helsinki, etc.) [2]. Researchers who submit dossiers to these committees in some countries most often complain about problems such as the complexity of the constituent elements required for the applications, the slowness of the analysis and delays in issuing authorizations, the inability to follow up on decisions, etc. Social scientists sometimes point out the impertinence of this mandatory pathway or the conditions under which dossiers are expected to be submitted [3, 4]. The committees we studied analyzed our application for approval to conduct socio-anthropological health research on the socio-cultural and community determinants of unwanted pregnancies and abortions in four countries: Peru, Palestine, Burkina Faso, and the Democratic Republic of the Congo (DRC). The empirical interest of this article lies in the fact that we considered four committees having studied the same dossier and that we actually experienced their functioning. We chose to do a comparative analysis based on the study of the functioning of these four ethics committees in the four countries. This involved observing the committees, their regulations, the nature of the certificates they issued, the constituent elements of the research approval applications, the work itself, the deadlines for analyzing the dossiers, and the conditions under which researchers then applied the committees’ recommendations. The core research questions are: What weaknesses of the committees explain the difficulties encountered by researchers? Are these weaknesses structural and general, or cyclical and specific to each committee? Studying the nature of these committees’ weaknesses, beyond the knowledge it provides, can help to improve their functioning and processing of applications, and to enhance researchers’ consideration of human rights. Our research was conducted using a qualitative approach.

## Methods

Document review, semi-structured interviews, and direct observations were the data collection techniques used. The first consisted of reading scientific articles and statutory and regulatory texts. The interviews, which were conducted between January 2015 and June 2016 in the four countries directly in local languages such as Mooré, Arabic, Lingala, and Spanish, were subsequently transcribed; the interviews in Mooré and Lingala were transcribed into French, the interviews in Arabic, into English. The interviews focused on the conditions for authorizing research, the dossiers to be submitted, the committees’ objectives, their functioning, their constraints, their results/efficiencies, etc. Respondents were members of ethics committees, men and women, with at least five years’ experience, general practitioners, gynaecologists, social scientists, lawyers, and community representatives. We conducted 47 interviews (10 researchers, 15 committee members, 20 managers and health services officers). Table 1 summarizes the specific elements in each country. Interviews generally lasted between 30 min and two hours; some respondents were interviewed more than once. The interviews were conducted by four data collection agents, including a senior researcher (the coordinator) responsible for organizing the survey in the country. The observations were indirect, in particular with regard to the timeliness of the committees’ services, communications among members during the dossier analysis, communication tools (websites, documents, etc.), certificates issued, and communications between committee members and requesting researchers.

**Table 1:** Documents required in dossiers for ethical approval of protocols, by country

Committee	Documents
National Ethics Committee for Health Research, Burkina Faso	1. Authorization request form duly completed and signed by the promoter 2. Research protocol 3. Researchers’ curriculum vitae 4. Information sheet and informed consent form 5. Declaration forms completed and signed by the researchers 6. Receipt for payment of the dossier review fees
Helsinki Committee Ethical Approval of Palestinian Health Research Council	1. Research protocol 2. Curriculum vitae of the principal investigator 3. Hand-written request for approval addressed to the committee chair
Institutional Ethics Committee for Humans of the Cayetano Heredia Peruvian University	1. Letter to the university’s research director 2. Declaration form completed by the principal investigator and the person in charge of operations at the institution where the research is to be conducted 3. Financial and conflict-of-interest declarations 4. Checklist of documents for the presentation of research projects 5. Research protocol and instruments to be used in the investigation (questionnaires, data collection forms, guides, etc.) and to obtain consent (informed consent, information sheets, etc.), with the date and version indicated on the protocol and on each instrument and consent form 6. Curriculum vitae of the principal investigator 7. Pay slip
Ethics Committee of the School of Public Health of the University of Kinshasa	1. Research protocol 2. Budget (for activities and investments) 3. Curriculum vitae for all the investigators 4. Informed consent form 5. Data collection questionnaire 6. Amendments 7. Copy of proof of funding 8. Progress report on activities/Annual report

Transcription of the interviews was entrusted mainly to the interviewers and coordinators, who had a complementary command of the different local and national languages. The national survey coordinators in Peru and Palestine spoke and wrote Arabic and Spanish fluently. The Arabic and Spanish texts were translated into French before being analyzed. The processing and analysis of empirical data were carried out using QDA Miner software, with the creation of codes and categories.

## Results

### Normative foundation for medical research ethics

On the international scale, there are 10 primary regulatory texts underpinning the creation of ethics committees: 1) the Nuremberg Code (1942); 2) the Universal Declaration of Human Rights (1949); 3) the Declaration of Helsinki on Ethical Principles for Medical Research Involving Human Subjects (1964, which has since undergone seven revisions, most recently in October 2013 in Fortaleza, Brazil; 4) the Belmont Report (1979); 5) the African Charter on Human and Peoples’ Rights (1981); 6) the International Ethical Guidelines for Biomedical Research Involving Human Subjects, prepared by the Council for International Organizations of Medical Sciences (CIOMS) (2002); 7) UNESCO’s International Declaration on Human Genetic Data (2003); 8) the Operational Guidelines for Ethics Committees that Review Biomedical Research, prepared by the World Health Organization (WHO) (2000); 9) the International Ethical Guidelines for Epidemiological Studies, prepared by the CIOMS in collaboration with WHO (2008); and 10) the Singapore Statement on Research Integrity (2010).

These texts provide the general basic principles upon which research ethics is founded. First, they address the person participating in research as either an actual or potential patient: the patient’s interest; the duty to treat and to protect the patient’s rights; the involvement of humans in research; respect for humans; the precedence of subjects’ rights over the pursuit of knowledge; the protection of subjects’ life, dignity, integrity, right to self-determination, privacy, and confidentiality; the possibility for all eligible groups of taking part in research; the increased potential for treatment provided to patients involved in research; and guaranteed compensation or appropriate treatments for subjects who have been harmed. These legal texts oblige researchers to obtain free and informed consent from the respondents, based on the objectives and the scientific and social results of the study. They also address utilitarian therapeutic knowledge (medical research aimed at producing health-related knowledge; the obligation for researchers to have research competencies), moral and regulatory policy issues (the obligation to respect national and international ethical standards), and impacts on environmental health (reduction of environmental pollution). The tenets of these texts are used in this article to analyze the committees’ practices with regard to monitoring researchers’ activities in the health field.

### Primary characteristics of the ethics committees

Beyond this malaise around fulfilling the primary objectives of ethics committees, there are unenforceable conditions in their texts (procedures, obligations, etc.) having to do not only with the analysis of protocols but also with the monitoring of surveys after they have been approved. With respect to analysis, we observed that almost no committee was able to assemble all members to analyze the dossiers submitted. In almost all the countries, ethical research standards are applied through an ethics committee whose mandate is to ensure researchers respect the standards set out in the texts listed above. Based on national regulatory recommendations inspired by the international declarations, these committees are structured to have the technical and organizational capacity to assess whether measures taken by researchers to respect the standards are actually applied. However, we noted that, in the four countries where we submitted research protocols for approval, the committees were all differently structured. Table 1 lists, for each country, the documents required by the ethics committees. The time frames, and the human and financial conditions associated with analyzing the submissions, are presented in Table 2. There are clear differences from one country to another.

**Table 2:** Committee structures and protocol review procedures, by country

Committees	Number of members	Number of items in the dossier	Processing fees	Turnaround time
National Ethics Committee for Health Research, Burkina Faso	9	6	70–800 EUR depending on who submits the request	2 months
Helsinki Committee Ethical Approval of Palestinian Health Research Council	3	3	No cost	1 week
Institutional Ethics Committee for Humans of the Cayetano Heredia Peruvian University	28	7	500 USD	4 weeks
Ethics Committee of the School of Public Health of the University of Kinshasa	15	8	500 USD	Unspecified

Source: Data from the documentary review, 2016

The lists of documents required by each country reveal fundamental differences in how ethical principles are taken into account. All committees considered the researcher’s competence and research quality to be important, as evidenced by their examination of the research protocol, the pay slip, and the curriculum vitae. On the other hand, some countries, such as Peru and Palestine, did not ask to see the informed consent form or any document that would enable them to monitor the interests of participant subjects. Whether a budget was submitted depended on each committees’ operational requirements. Only the DRC required a research progress report in the dossier.

### The National Ethics Committee of Burkina Faso

Burkina Faso’s Health Research Ethics Committee (CERS) was created by Decree no. 2002-536/PRES/PM/MS/MESSRS dated November 21, 2002, which specifies in its Article 2 that it is “a decisional body mandated to oversee the respect of the principles set forth in the National Code of Ethics with respect to health research. It is independent” [author’s translation]. It is supplemented by Joint Decree no. 2004-147/MS/MESSR of May 11, 2004, which delineates the organization and functioning of the Health Research Ethics Committee in Burkina Faso [5]. Its mission is to analyze protocols to ensure the respect of ethical standards, as well as to make recommendations [6]. The committee has nine members, whose mandates are for three years and renewable. It is made up of three representatives of the Ministry of Health, two representatives of the Ministry of Research and Higher Education, and one representative each from the Ministry of Animal Resources, the Ministry of Human Rights, the Order of Physicians, and the Order of Pharmacists. The Committee reports to the Ministry of Health. The application package consists essentially of the protocol with research tools, briefing note, researcher resumes, consent forms, and research budget.

The process of analyzing and approving our protocol took around one month; the official turnaround time for approval is two months. One committee member with whom we were able to meet provided the following information on its functioning:

To assess research protocols, the CERS meets monthly at regular sessions convened by the chair. When necessary, extraordinary meetings can also be convened either by the chair or by one-third of the members. The chair convenes the meetings and leads the discussion. It is also he who delivers the ethics certificates. The committee can deliberate validly only if at least two-thirds of the members are present. Meetings are held in camera, and decisions are taken by consensus; failing that, an absolute majority of the members is required. The committee is required to come to a decision within two months of receiving a request and, when the ruling is positive, the chair delivers an ethics certificate within 15 days of the decision. In the absence of a code of ethics for health research, the CERS refers to various national standards, in particular the codes of ethics of various bodies of health professionals and the advice of other experts, but mainly it consults international standards in this matter. Finally, it should be noted that it is the Ministry of Health that provides, to the extent possible, the material and financial means required for the CERS to carry out its mandate. The rulings of the CERS are compiled in a report sent annually to the minister responsible for health.

### The Helsinki committee ethical approval process, University of Gaza in Palestine

In Palestine, the Palestinian Health Research Council houses the Helsinki Committee Ethical Approval process. Our protocol was analyzed by three members (the chair and two other members), whereas the available information states that this is a committee of 10 members made up of physicians, hospital administrators, and paramedical officers. We were not given any information to the effect that a dossier might be analyzed by only a few members, but in some cases dossiers could be reviewed by a smaller committee when the research project is considered to entail very low risks for participants. The committee, consisting of 10 members, was created in 1980 and its foundational texts were revised in 1999. The dossier for the research approval request consists of a letter from the research director, proof of registration (payment of fees), the research director’s confidentiality compliance statement, the statement from the institutional manager in the research operational area, the declaration of conflicts of interest, the researchers’ resumes, and the research protocol. Our dossier was analyzed within two weeks. The approval we received was valid for two years. There was no charge for reviewing the dossier. The members stipulated, as a general condition, the requirement that they be informed of any change in the protocol. There was no requirement to submit any report during the period of validity of the approval. Once the approval was granted, we had no further obligations toward the committee.

### The institutional ethics Committee for Humans of Cayetano Heredia Peruvian University

In Peru, committees are generally attached to the universities. Our experience was with the Institutional Ethics Committee for Humans (CIEI) of Cayetano Heredia Peruvian University (a private university) in Lima. Independent both financially and administratively from the university, it is charged with “protecting the rights, the well-being, and the safety of research participants. In research conducted with humans, the CIEI ensures that ethical responsibilities and national and international regulations are respected” [author’s translation]. Created in June 1992, the committee is composed of 29 members, of whom eight are from outside the university. The eight external members include one lawyer, two doctors, and five others whose profiles are not defined. They represent the community. The other 21 members are from the university. Members include physicians, psychologists, anthropologists, and nurses, among others. The committee meets every two weeks to analyze pending dossiers.

The CIEI is an autonomous committee formed by a multidisciplinary team of highly qualified professionals and of esteemed members of society (http://www.upch.edu.pe/vrinve/duict/regulacion/etica/cieh.html). It meets every two weeks. The following elements are required when submitting a research project:

- completed CIEI form;
- research protocol;
- informed consent instruments (for adults, adolescents, and parents of minors);
- resumes of the principal investigators.

Once the dossier has been submitted and the fee of 450 USD paid, the turnaround time for a response from the committee is four weeks. Research projects are approved for 12 months. Once the project has been approved, researchers must submit progress reports after six and 12 months, as well as when the project is completed or a renewal of approval is sought. The ethics committee reserves the right to monitor the terms of signed informed consent in the case of any problems.

At the CIEI, the items that make up the dossier vary depending on the purpose of the study. Nevertheless, the basic components are: the letter of submission addressed to the Chair; a form to be completed indicating the types of participants, the budget, the project start and end dates, its duration, the types of medical procedures involved in the study, the risks and benefits for participants, the recruitment process, compensation, confidentiality, anonymity, etc.; the original research protocol; instruments for obtaining informed consent (for adults, adolescents, and parents of minors); and the resumes of the principal investigators. These documents are supplemented by others (See Table 1 for the exhaustive list of documents required when submitting a study protocol) depending on whether the research is a clinical trial or some other type of study. For example, proof of funding to cover potential damages is required. The cost of processing the dossier is 450 USD, regardless of the budget of the research project.

The final report is a key component in any request for renewal of the approval at the year’s end. In the case of such renewal requests, the ethics committee may monitor the documents completed and signed by study participants. For our study, it took eight weeks to receive approval.

### Ethics Committee of the School of public health of the University of Kinshasa, DRC

In the DRC, the national bioethics committee was created in 2004. Its mission is to “give the go-ahead to the start of a study, to conduct ongoing review of the study, and to propose to the Ministry of Health the cessation of a study” [author’s translation] [7]. The committee is made up of 15 members, which include ex officio members (pharmaceutical advisor to the Ministry of Health, medical advisor to the Ministry of Health, president of the Order of Pharmacists, vice-dean of research of the Faculty of Pharmacy of the University of Kinshasa, vice-dean of medicine of the Faculty of Pharmacy of the University of Kinshasa, president of the Order of Physicians, director of the National Biomedical Research Institute) and members appointed by groups (Non-Governmental Organizations (NGOs), high-risk groups, media, lawyers, educators, pastors, and police). The members’ mandates are for five years, renewable once. Members receive an allowance, based on a rate set by the Ministry of Health, under advice from the committee [6]. The dossier for a request for approval consists of the research protocol, the questionnaire, the informed consent form, the researchers’ resumes, the budget, amendments previously obtained, progress report on activities, and proof of payment of fees. The dossier is to be submitted in 10 copies.

Table 2 summarizes the structures and procedures for the ethics committees in the four countries.

### Structural and organizational differences among the ethics committees

Committee functioning has to do with composition and member structure, turnaround time, processing fees for analysis of dossiers, institutional affiliation, and items to be included in the dossiers. These parameters differ from one country to another. This is especially the case for turnaround times, which depend on a variety of people and institutions.

Looking at this table, we see that the committees’ functioning and structures are quite varied. The DRC’s committee, which actually only began functioning in November 2013, has 15 members, including physicians, sociologists, anthropologists, etc. Burkina Faso’s committee has nine members, while in Peru, the committee has 29 members.

Turnaround times for approving dossiers range from two weeks to three months depending on the committees. These times are set in their regulations. While some committees manage to respect those times, others do not. In our case, the Helsinki Committee Ethical Approval process of the University of Gaza was the most prompt, at just one week. Approvals from Burkina Faso, the DRC, and Peru took one month, six weeks, and two months, respectively. For Burkina Faso, the approval granted at the national level did not directly authorize us to begin observations and interviews in the field. Rather, it opened the door for us to submit a second request, this time to the regional departments involved, for operational authorization. As a regional officer explained:

“In our view, authorities who are very high up in the hierarchy cannot issue an operational authorization. It is up to the regional departments to verify more closely the conditions of the survey. Our authorization is more determinant than that of the national authorities, in terms of ensuring the respect of ethical norms.” [Manager, Regional Health Department, Burkina Faso, November 2015]

As such, obtaining approval here requires several more days or weeks. This particular arrangement was only seen in Burkina Faso. The situation was very different in Palestine. The committee, located in the University of Gaza and coming under the Palestinian Authority, has no regional local equivalents. The national administrative organization being in a context that is both quite Islamized and under the Israeli protectorate does not lead to a great deal of decentralization of ethical matters. As such, ethics committee members are far removed from the researchers’ social context. This explains many of the difficulties encountered, as explained in the following excerpt:

“It was not easy to meet with the chair of the ethics committee, even to submit the dossier. Everything was done by mail. We have no technical reference for the committee in terms of social or administrative location, except for the university. Fortunately, the committee does not take long to respond to requests submitted.” [Medical referral officer, Gaza, September 2015]

In Peru and the DRC, the committees are housed in the universities, with the DRC’s being in the School of Public Health and Peru’s in a private university and among 50 other committees at the national level. The fact that these committees, like that of Gaza, are located in universities, means there is no local representation, as there is in Burkina Faso, whose committee is located with the Ministry of Health. To some extent, this institutional position was sometimes helpful for our request in terms of turnaround time, as illustrated by the comments of one researcher at the University of Kinshasa:

“I know several members of the ethics committee. They often don’t have much time. But I’ll make sure your approval is issued as soon as possible. Just make sure you provide all the documents required.” [Teacher, University of Kinshasa, October 2015]

Then, fees for analyzing the dossiers are also variably applied. While there were no fees in Gaza, the fees were about 400–500 EUR in the other committees. The method of calculating this cost is, in general, not very rigorous. Researchers submitting a request for approval might modify the actual budget for the study to present a lower amount; in some countries, the fees are calculated based on that amount. Most often, committee members are flexible and open to negotiating the fees in terms of the regulations. This was the case in the DRC, where we were able to obtain a colleague’s assistance in lowering the cost of processing our request.

Finally, as shown in Table 1, the number and composition of the documents to be included in the ethics committee dossier also vary from one country to another. While Gaza requires only the protocol, DRC, and Peru ask for around 10 documents. Depending on the committee, 10 to 15 copies of each document are required. In Peru, the four documents are to be submitted in one copy only.

### Structural and organizations similarities among the ethics committees

Points of similarity among the ethics committees included multidisciplinary membership, institutional affiliations of the members, the objectives, and the difficulties of mobilizing the members for meetings to analyze the dossiers submitted.

First, for all the committees, members were from different professions and institutional affiliations. In general, they included academics (physicians, anthropologists, sociologists, psychologists, philosophers, etc.), non-university hospital personnel, lawyers, members of associations, etc. Members represented either researchers or the communities in which research is conducted. Whereas the former worked to develop their corporate base, the latter worked to protect the interests of their base.

For example, “the CIEI is an autonomous committee established by the Cayetano Heredia Peruvian University. It is formed by a multidisciplinary team of highly qualified professionals and esteemed members of the society. Its objective is to protect the rights, well-being, and security of research participants. The CIEI ensures compliance with ethical responsibilities and with national and international regulations with respect to research conducted with humans.” (CIEI member, Lima, November 2015).

Then, of the four committees studied, three are housed in universities. The committee in Burkina Faso, which is located in the Ministry of Health, has academics among its members. The relationship with the university is closely linked to the nature of the committees as bodies supporting research. As such, they are “morally and technically” grafted to universities.

Finally, in all the countries, our dossiers were analyzed by a subset of the members; it is very difficult for a committee to convene all the members to analyze a dossier together. On this subject, a committee chairperson made reference to the heavy professional workloads of members, in this case academics, their volunteer status, and their conflicting schedules.

“In order for the committee to carry out its activities of analyzing dossiers, the chairman always has to make do with just a few of the members. It is routinely difficult, if not impossible, because of professional workloads, conflicting schedules, and the volunteer nature of this function, to bring together all the members as envisioned.” (Committee member interview, Peru, December 2015)

### Structural weaknesses common to all four ethics committees

The procedures for the ethical review systems all exhibit certain fundamental and cross-cutting weaknesses. These include the lack of monitoring to ensure researchers respect the standards, the non-evaluation post-research of the impacts on participants, and the social and regulatory distance between participants and the institutions charged with protecting them.

First, we learned from the interviews that none of the committees was capable of verifying whether the dignity and security of the study participants were respected, in accordance with the foundational texts on ethical principles for research presented earlier in this article. Citing insufficient resources of all types, the committees fail to carry out this fundamental and primary purpose for their existence.

Their capacity is limited to studying and approving dossiers. Yet, according to the chairs and institutional members we interviewed, they are under obligation to monitor the surveys for all protocols they approve. In Burkina Faso, Article 25 of the Joint Decree stipulates that the ethics committee can organize visits to assess or audit the implementation of research protocols, but does not specify whether this is sanctioned by a minuted report of the visit and, if so, to whom that is to be addressed. It is only for biomedical research, as presented on the website of the Ministry of Health, (http://elearning.trree.org/mod/nationalsupplement/view.phpid226&langfr), accessed June 30, 2016 that the situation is somewhat clearer. In fact, Article 13 of Decree no. 2010-292/MS/CAB on the conditions for granting authorizations for clinical trials stipulates that “any duly authorized clinical trial may be subject to inspections by services designated by the Ministry responsible for health to ensure respect of the protocol’s application. An inspection report can recommend legal proceedings and the suspension or immediate cessation of any clinical trial in cases where norms are not respected” [author’s translation].

After approval, there is no follow-up to ensure the recommendations or even the research programs contained in the dossier submitted have been applied, according to interviews with the health service officers and the committee members. This may be due either to a lack of resources or to the material unavailability of the members, whose roles in the committee are secondary to their professional occupations. In this respect, all the committees were alike.

“We have neither the material nor the financial means, nor even the time, to go out in the field to monitor whether researchers are respecting ethical standards.” [Regional health director, Burkina Faso, November 2015]

Then, the committees overall are not able to monitor whether the researchers requesting authorization actually fulfill the written commitments made in their requests. We experienced this concretely in the course of our study. How to ensure that the researchers have implemented informed consent, or confidentiality? How to ensure that, years after the trial or study, the researchers have been able to maintain confidentiality regarding the serological status of the persons they interviewed? This problem is part of a wider issue of the judicialization of the public health service in general. Indeed, even though the obligations of care provision are different from those of research, we did not find in our research any cases of actual complaints against health workers for medical errors. If there are no user complaints about medical errors, which are more common, then it is likely that errors related to confidentiality among research participants are even more rare. We can reasonably conclude there is not yet sufficient public awareness about researchers’ responsibility regarding the products of research.

“Here, the chair of our committee is a religious dignitary, a bishop. Because of this, he is especially demanding when it comes to a dossier dealing with unwanted pregnancies and abortions. But once the project is approved, he doesn’t have time to check on how the study was conducted. If there are no complaints from participants, there will be no problem.” [Anthropologist-researcher, Peru, October 2015]

Moreover, committee members were often unable to assess the implementation of protocols. Without being normative, it is clear that, to gain a better understanding of the “practice standards” [7] of researchers who might deviate from the committees’ official standards, committee members would have to conduct occasional field visits during the survey.

“Given the heavy workload of each of the key members of the committee, we clearly don’t have time to monitor the surveys. Also, unfortunately, once the researchers have obtained approvals, they don’t send us the required progress reports. We have to rethink how the ethics committee’s requirements are operationalized.” [Ethics committee member, DRC, October 2015]

The following statement by the chair of the committee at the School of Public Health of the University of Kinshasa is indicative of strategic and operational malaise. Although the committee was created in 2006, it was only able to be installed in 2013. In an interview in 2014, the chair stated:

“Today, we’re still in the phase of training all the members. But once that’s done, we’ll be able to fulfill the missions we’ve been assigned. This committee’s first vocation is to provide advice or make reports to government and to propose legislative texts. It’s also supposed to work on improving physician training in the field of ethics and on raising awareness on this matter among patients and the general public. One priority will be, for example, to set up clinical ethics structures in all hospitals to reflect on very concrete issues relating to the ethics of healthcare.”

### The difficulty of obtaining free and informed consent

A fundamental objective of ethics committees is the obtention of participants’ free and informed consent prior to participation in field surveys. The experience in our own survey showed us that committees fail to achieve this principle for two reasons: patients’ fear of the risk of being exposed and the power of health workers.

Indeed, on the ground, particularly in Gaza and Lima, we experienced great difficulty in being able to meet with women who had experienced unwanted pregnancies. In Gaza, we met with five women, all of whom were managers of health services or associations. Yet NGOs and health services insisted that the phenomenon of abortion of unwanted pregnancies existed and was widespread. Given the sensitivity of the subject, no girl or woman would be willing to make herself known at the risk of reprisal. As one 25-year-old female physician in Gaza confided to us, “At this age, not being married by now, I’m sure I’ll never marry. I’m outside the limits of what is tolerable here in Gaza.” (Survey interview, Gaza Strip, November 2015). The case of the DRC is sometimes to the contrary, but still not ideal in terms of informed consent. Indeed, given the scale of the situation of unwanted births and clandestine abortions, women had no problem making themselves known. We encountered young women with two to three children born before marriage and of different fathers. In this overall situation, and because of the social impact of the phenomenon, women confided readily, with the aim of obtaining humanitarian assistance. Their consent is often not linked to the scientific objectives of the application. They are hoping for direct aid that will help them to solve everyday problems.

On this issue and in Burkina Faso, we observed a kind of expression of health workers’ and NGOs’ power of intervention in development. Given our insistence on meeting women with an unwanted pregnancy experience, and under cover of our sponsor, Médecins du Monde France, health workers (midwives, maternity nurses, etc.) “negotiated” [8] with these women to “consent” to be interviewed by us. In this way, we were able to obtain interviews. Yet despite this, in certain cases, we did not get direct testimony from the woman. Unconvinced of our confidentiality, some women spoke not in the first person but in the third person singular: “I’m telling you about the experience of acquaintance; she has...”.

A difficulty with respect to the application has to do with ensuring the authenticity of patients’ consent to participate in the survey. In fact, the requirement calls for patients to sign the consent form. In many, if not most, cases participants are reticent to sign. Given this fear of signing, it has been suggested that patients’ consent be recorded when they accept to be included in the source population for the study. However, this approach also has its shortcomings. Contrary to the basic intent, which is to persuade participants of the value of the research and the good faith of the researchers, asking for their signature makes them think they will be held responsible for what they say and could be taken to task for it. Consequently, during our experience, the four ethics committees presented concerns about the management of obtaining free and informed consent.

Finally, the teams mandated to apply ethical standards are not able to grasp the situations of participants in certain contexts, generally in the case of research involving patients. In fact, illness either attracts or repels, depending on whether it is considered natural or mysterious [9], or depending on the social characteristics of the ill person. Many illnesses, especially those due to non-tolerated social practices, can lead to actual or self-imposed isolation. AIDS, for example, as well as different forms of hepatitis, cancer, and sexually transmitted diseases, do not easily allow for social integration with the identity of the illness.^1^ Participants’ real or imagined rejection, and the lack of community or public institutions to defend their interests, especially in the South, also explain why committees have difficulty establishing communications with them. In accordance with fundamental ethical principles—the precedence of subjects’ rights over the pursuit of knowledge, and the protection of subjects’ life, dignity, integrity, self-determination, privacy, and confidentiality—the committees, whose members are generally involved professionally in both care and research—should strive to institutionalize and maintain patients’ social integrity in the most discriminating and high-risk cases.

### A universal project caught in a contextual net?

In our study, we observed that ethics committees have different structures. Their members are drawn from very different institutional and professional sources. Turnaround times for analyzing dossiers and responding to researchers’ requests are also very different. In general, the times are longer for committees that try to work as a team. Committees with shorter turnaround times are often led by one or a few individuals endowed with particular authority. The processing fees are also calculated in different ways. For some committees, processing is free of charge.

Despite having the same philosophical and disciplinary origins, the ethics committees’ structures and functioning differ from one country to another.

Beyond this malaise around fulfilling their primary objectives, there are unenforceable conditions related to both the analysis of dossiers and the operationalization of the studies. We observed that almost no committee was able to convene all members for analysis of the dossiers submitted. In general, committee members are people with heavy workloads, given their profiles and their functions. They therefore have difficulty making themselves available to review dossiers for approval. Even when they attend meetings for certain projects, they do not always have time to analyze the dossiers seriously and to determine whether the conditions generally considered essential are actually present [9, 10].

## Discussion

The following key points emerged from analysis of the results regarding the four ethics committees: generalized ineffectiveness in terms of protecting the interests of human research subjects; differences in structure and functioning; and difficult institutional implementation with respect to research locations (universities). This latter point is at the core of some fundamental structural problems.

Indeed, a literature review showed that research ethics committees in sub-Saharan Africa have major shortcomings such as “lack of membership diversity, scarcity of resources, insufficient training of members, inadequate capacity to review and monitor studies, and lack of national ethics guidelines and accreditation” [11]. All these shortcomings contribute to the weak achievement of these structures’ fundamental objectives, as has been observed in committees, even in continents where they are more long-standing and where resource problems of all kinds are sometimes less severe.

First, difficulties in achieving fundamental objectives, such as protecting the interests of survey participants, have been raised elsewhere. Researchers interviewed after having submitted requests for protocol approval questioned these committees’ effectiveness. Most committees do not have the means to achieve their core objectives [12, 13]. Burris and Moss (2006) believe it is not a question of professional conscience and ethical sensitivity among researchers, but rather a problem of regulating even norms [12]. Numerous problems have been raised with regard to how these committees are managed and how they monitor implementation of their recommendations [1, 11].

Second, the issue of differences in structure, and even more in operations and decision-making, has been examined elsewhere, as in the United States [14, 15]. Variations in approval times have been observed, ranging from five to 172 days [16]. These differences, attested to by several researchers, have been attributed not to differences in the researchers’ communities of origin, but rather to factors related to the committees, which are very subjective, as the following quote indicates.

“Differences often persist because Institutional Review Boards (IRBs) think these are legitimate, and regulations permit variations due to differing “community values.” Yet, these variations frequently appear to stem more from differences in institutional and subjective personality factors, and from “more eyes” examining protocols, trying to foresee all potential future logistical problems, than from the values of the communities from which research participants are drawn. However, IRBs generally appear to defend these variations as reflecting underlying differences in community norms” [12].

Third, issues of form (in relation to the content of the various documents) and of substance—in particular, standards to be respected and rigidity in compliance with them—have also been examined with unsatisfactory conclusions [17, 18]. On this issue, a study, also in the United States, showed that ethics committees are often embarrassed when it comes to evaluating the scientificity of the protocols submitted to them. This embarrassment is, in fact, the expression of a technical and operative incapacity of the material systems supporting their operations.

In view of this literature, abundant in North America, the structural and organizational differences among ethics committees, as well as their main weaknesses and failures in achieving their humanist ambitions, are almost universally acknowledged.

## Conclusions

Based on the same ethical principles and operating on the basis of fundamentally identical regulatory texts, the ethics committees we analyzed in four different countries (Burkina Faso, DRC, Palestine, and Peru) straddling three continents (Africa, America, and Asia) generally have very significant difficulties in terms of their functioning and, above all, in achieving the objective of genuine respect for human dignity. Indeed, researchers, who are operationally distant from the committee members, are still unable to achieve the understanding needed for real consent. In almost all countries, however, committee membership remains voluntary; committees are not funded. As such, they lack the means to effectively enforce regulatory and statutory requirements.

Our study noted significant institutional differences between ethics committees. While in DRC, Peru, and Palestine, the committees are run by universities on a local scale, in Burkina Faso the committee is situated with the Ministry of Health, with national coverage. These institutional differences may explain discrepancies in their capacity to deliver the intended services to users.

Our study of the four above-mentioned ethics committees showed that several problems are handled differently by these committees.

Having concluded our analysis, we raise three questions for consideration. What is the most appropriate organizational and statutory model for institutional mechanisms to regulate ethical standards in research in the social sciences of health? What value is added by such mechanisms in terms of the dignity of participants in those countries? What institutional and community-based response (from the participants’ standpoint) would make these mechanisms more effective?

Based on the cases we analyzed, we consider that a simple structure like that of Gaza in Palestine is best able to provide a timely response to researchers’ requests for approvals. For a committee to successfully manage the other challenges—particularly having close contact with participants, monitoring the application of protocols, and doing post-operational evaluations—requires a core group of remunerated executive members. Given the current status of these committees’ institutional and operational mechanisms, there is very little added-value [12, 19]. For participants to derive the greatest benefit from these committees, there must be a place where they and committee members can meet and exchange views. For this, protocols must be set up to identify representatives of target groups with whom communications can be maintained throughout the studies.

From our experience we can venture the provocative opinion that health research ethics committees, which are relatively unknown and poorly supported by public authorities, are having difficulty carving out a place for themselves in terms of becoming standardized and conducting real work. They limit themselves to satisfying the requirements of bureaucratic formalities for project leaders, even though they do not have the means to conduct verifications afterwards. Most often, their cumbersome and sluggish approach to organizing the review of dossiers delays the implementation of studies. While they are intended to protect the dignity, respect, and integrity of service users in general and research participants in particular, they are burdened with material and financial constraints that prevent them from fulfilling their mandates.

## Abbreviations

CERS: Health Research Ethics Committee (Burkina Faso)
CIEI: Institutional Ethics Committee for Humans of Cayetano Heredia Peruvian University, Lima, Peru
CIOMS: Council for International Organizations of Medical Sciences
DRC: Democratic Republic of the Congo
IRBs: Institutional Review Boards
LASDEL: Laboratoire d’Etudes et de Recherche sur les Dynamiques Sociales et le Développement Local
NGOs: Non-Governmental Organizations
WHO: World Health Organization

## Footnotes

1 It is important to note that, in countries such as France, patients have banded together to defend their healthcare rights, but in Southern countries this movement is still marginal.
